# Risk assessment for Thai population: benchmark dose of urinary and blood cadmium levels for renal effects by hybrid approach of inhabitants living in polluted and non-polluted areas in Thailand

**DOI:** 10.1186/1471-2458-14-702

**Published:** 2014-07-09

**Authors:** Muneko Nishijo, Yasushi Suwazono, Werawan Ruangyuttikarn, Kowit Nambunmee, Witaya Swaddiwudhipong, Kazuhiro Nogawa, Hideaki Nakagawa

**Affiliations:** 1 Department of Public Health, Kanazawa Medical University, 1-1 Daigaku, Uchnada, 920-0293 Ishikawa, Japan; 2 Department of Occupational and Environmental Medicine, Graduate School of Medicine, Chiba University, Chiba, Japan; 3 Division of Toxicology, Faculty of Medicine, Chiang Mai University, Chiang Mai, Thailand; 4 School of Health Science, Mae Fah Luang University, Chiang Rai, Thailand; 5 Department of Social Medicine, Mae Sot General Hospital, Mae Sot, Tak Province, Thailand

**Keywords:** Cadmium, Benchmark dose, Urinary cadmium, Blood cadmium, Renal effect

## Abstract

**Background:**

The aim of the present study was to estimate the benchmark doses (BMD) for renal effects for health risk assessment of residents living in Cd-polluted and non-polluted areas in a Thai population.

**Methods:**

The study participants consisted of inhabitants aged 40 years or older who lived in a non-polluted area (40 men and 41 women) and in the environmentally polluted Mae Sot District (230 men and 370 women) located in northwestern Thailand. We measured urinary and blood cadmium (Cd) as markers of long-term exposure and urinary β2-microglobulin (β2-MG) and N-acetyl-β-D-glucosaminidase (NAG) as renal tubular effect markers. An updated hybrid approach was applied to estimate the benchmark doses (BMD) and their 95% lower confidence limits (BMDL) of urinary and blood Cd for Cd-induced renal effects in these subjects. BMD and BMDL corresponding to an additional risk (BMR) of 5% were calculated with the background risk at zero exposure set to 5% after adjusting for age and smoking status.

**Results:**

The estimated BMDLs of urinary Cd for renal effect markers were 6.9 for urinary β2-MG and 4.4 for NAG in men and 8.1 for β2-MG and 6.1 for NAG μg/g creatinine (Creat) in women. These BMDLs of urinary Cd (μg/g Creat) for NAG were less than the geometric mean urinary Cd in the polluted area (6.5 in men and 7.1 in women). The estimated BMDLs of blood Cd (μg/L) were 6.2 for urinary β2-MG and 5.0 for NAG in men and 5.9 for β2-MG and 5.8 for NAG in women. The calculated BMDLs were similar or less compared with the geometric mean blood Cd (μg/L) in the polluted Thai area (6.9 in men and 5.2 in women).

**Conclusion:**

The BMDLs of urinary and blood Cd for renal effects were estimated to be 4.4 - 8.1 μg/g Creat and 4.4 - 6.2 μg/L in the Thai population aged ≥ 40 years old, suggesting that more than 40% of the residents were at risk of adverse renal effects induced by Cd exposure in Thailand.

## Background

In the Mae Sot district, Tak Province, northwestern Thailand, serious cadmium (Cd) contamination of soil and rice due to river water contamination suspected from upstream mining activity was reported by the Thai Ministry of Agriculture in 2003. In the polluted area, more than 90% of the rice grain samples were contaminated with Cd that was greater than 0.2 mg/kg (ppm), the recommended level by the European Union [1], and 85% of the surveyed paddy soil samples had a Cd concentration that was greater than 3 ppm, the recommended level by the Codex Committee [2]. A population screening survey for cadmium exposure using urinary cadmium measurement was conducted in 2004 among inhabitants aged 15 years and older living in these cadmium-contaminated villages. Of the 7,697 adults examined, 7.2% had urinary cadmium levels ≥5 μg/g creatinine (Creat) [2], and the prevalence of renal dysfunction (defined as urinary β_2_-microglobulin (β2-MG) ≥1,000 μg/g Creat) was found to be 19.9% among 527 persons with urinary Cd > 5 μg/g Creat [3].

The benchmark dose (BMD) method has been adopted to evaluate the health risks of environmental contaminants [4,5]. The BMD is defined as the exposure level that corresponds to a specific increase in the probability of an adverse response (benchmark response, BMR), compared with the response at zero background exposure. The lower 95% confidence limit of the BMD (BMDL) can be used in risk assessment as a replacement for the no observed adverse effect level (NOAEL) [4,5]. For an index of Cd exposure, urinary Cd concentration has been adopted because it is mainly influenced by the body burden of cadmium and is proportional to the concentration in the kidney [6]. Consequently, as a health risk assessment of Cd exposure, several studies in the Japanese population have applied the BMD method to the relationship between renal effect markers and urinary Cd as an index of internal body burden [7-11]. Recently, estimations of BMD and BMDL for continuous outcomes using the hybrid approach have been developed [12,13]. Using this method, the BMD and BMDL were estimated based on continuous exposure and a continuous effect marker, thereby avoiding categorization of the subjects [13,14]. Accordingly, the statistical validity and efficiency of the BMD and BMDL values were greater using the hybrid approach compared with methods involving categorization of continuous exposure and effect markers. By using this hybrid approach, the BMDL of urinary Cd for renal effect markers in the Japanese [15-18] and Swedish population have been estimated [19]. However, the relationship between urinary Cd and renal dysfunction has not been evaluated in the Thai population.

Typically, BMD and BMDL estimations based on the hybrid approach have been conducted in previous studies for urinary Cd, and not for blood Cd. This is because blood Cd has not been considered the most valid marker of Cd body burden, but recent exposure [6,20]. However, the high correlation between urinary Cd and blood Cd observed in previous studies [6,21,22] indicates that blood Cd is thought to be a good estimates of cadmium body burden in environmentally exposed populations whose Cd exposure was consistent. Furthermore, the urinary excretion route of Cd is the same as with other urinary substances including renal effect markers, and there is some concern about the effects of other substances in urine on the relationship between urinary Cd and renal dysfunction. Particularly, targeted Thai subjects in the present study showed a high prevalence of urinary tract stones [23], and significant increase in urinary β2-MG related increased urinary calcium (Ca)/citrate, a stone-forming risk factor correlated with urinary Cd [24].

Therefore, the aim of the present study was to apply an updated hybrid approach to estimate BMD and BMDL of Cd exposure for Cd-induced renal effects in a Thai population using both urinary and blood Cd as exposure markers of Cd.

## Methods

### Study subjects

The study participants consisted of inhabitants aged 40 years or older who lived in a non-polluted area in the same district (40 men and 41 women) and in the environmentally polluted Mae Sot District (230 men and 370 women) located in northwestern Thailand. The subjects in the polluted area were participants aged ≥ 40 years in the 2007 health survey (participant rate 83.7%) targeted for residents whose urinary Cd levels were ≥5 μg/g Creat in 2004–5 [25,26]. The numbers of subjects in the non-polluted area was small, which is a serious limitation of this study, but addition of these subjects in the non-polluted area increased the low rate of subjects with urinary Cd < 5 μg/g Creat in the polluted area.

These areas were rural, and their socioeconomic environments were similar. Each participant was interviewed by well-trained nurses about demographic characteristics, smoking status, alcohol consumption, and their medical history of chronic diseases. The subjects were requested to provide urine samples and fasting venous blood samples for biological measurements. The present study protocol was approved by the Ethical Committee of Chiang Mai University in Thailand and Kanazawa Medical University in Japan, and written informed consent was obtained from all subjects prior to participation after explanation of the survey by the medical doctor in Mae Sot General Hospital, one of the authors in the present study.

### Sample measurements

A urine sample from each subject was divided into three aliquots (3–5 ml each), and all aliquots were frozen and stored at -20°C for later analysis. Prior to storage, one drop of 0.5 N sodium hydroxide was added to one of the three aliquots of urine showing a pH of 5 or less to adjust the pH to 6–8 to prevent further degradation of β2-MG in acidic conditions.

Blood and urinary Cd concentrations were quantified using a flameless atomic absorption spectrometer (Shimadzu Model AAS-6300, Kyoto, Japan). The urine was diluted with 20 mg/l palladium chloride solution in 5% HNO_3_ as a matrix modifier at a ratio of 1:1. Proteins in blood were precipitated by 5% HNO_3_ at a ratio of 1:2 [26]. Method validation of the technique was performed and verified using certified standard reference materials [urine reference material Lot No. 2670 (National Bureau of Standards, Washington D.C., USA) and control blood Lot No. 620302 (Behring Institute, Dresden, Germany)] to ascertain the accuracy and precision of the method. Detection limits of urinary and blood Cd were 0.06 μg/g Creat and 0.2 μg/L, respectively. The urinary Cd of each subject was adjusted for urinary Creat concentration, which was measured using an enzyme assay Cica liquid–S (Kantokagaku Reagent Division, Ltd., Japan).

Urinary β2-MG was measured by enzyme immunoassay using a latex agglutination immunoassay (Eiken Chemical, Japan). Urinary N-acetyl-β-D-glucosaminidase (NAG) concentration was determined by a colorimetric assay using a NAG test kit (Shionogi Pharmaceuticals, Japan). Concentrations of both urinary NAG and β_2_-MG were also adjusted per g Creat.

### Statistical analysis

We used the maximum likelihood approach to fit the dose-effect model to the data [12,13]. To obtain a symmetrical distribution, data on β2-MG and NAG were natural log-transformed. The model for the mean response, *μ*(*d*_*i*_), was assumed to be linear:

(1) $$\mu \left(d_{i}\right)=\beta_{0}+\beta_{1}\times d_{i}$$

Where *d*_*i*_ = dose for the i-th individual. Urinary Cd or blood Cd, age and smoking status were included in the statistical model and analyzed using multiple linear regression analysis. For urinary and blood Cd levels, Akaike Information Criterions (AICs) of the quadratic, cubic, or logarithmic model were similar or increased compared with that of linear model, and regression coefficients of the quadratic and cubic terms were not significant; therefore, we used the linear model in the present study.

The BMDs and BMDLs were calculated using the hybrid approach, which allows for the calculation of risk of continuous data without dichotomizing the outcome [13,14]. The BMR was defined as a 5% additional risk because a BMDL corresponding to an additional risk of 5% was close to the NOAEL on average [27]. For positive associations between exposure and renal effects, the effect level associated with a certain BMR equals:

$$\mu \left(\mathrm{BMD}\right)=\mu \left(0\right)+\sigma \left(\Phi^{‒1}\left[1‒P\left(0\right)\right]‒\Phi^{‒1}\left[1‒P\left(0\right)‒\mathrm{BMR}\right]\right)$$

Where *σ* = the standard deviation of residuals, Ф^-1^ = the inverse of the standard normal cumulative distribution function, and *P*(0) = the background probability of a response defined in terms of a specified tail proportion of a “hypothetical” control distribution (at urinary/blood Cd = 0), which in this study was set at 5%. The corresponding continuous cut-off values, *c*, for specified values of renal effect markers in terms of *P*(0) are given by:

$$c=\mu \left(0\right)+\sigma \times \Phi^{‒1}\left[1‒P\left(0\right)\right]$$

The BMD was calculated by combining the equation for *μ*(BMD) with that of the dose–response model [1]:

(2) $$\mathrm{BMD}=\frac{\sigma }{\beta_{1}}\times \left(\Phi^{‒1}\left[1‒P\left(0\right)\right]‒\Phi^{‒1}\left[1‒P\left(0\right)-\mathrm{BMR}\right]\right)$$

The BMDL (defined as the one-sided lower 95% confidence limit of the BMD) was calculated as representative reference exposure using the profile (maximum) likelihood method, which can be used to compute confidence intervals [12,13].

In addition, we observed that this approach did not require the actual reference population to determine the cut-offs, which may have had a large influence on the estimation of the BMDL [19] because we defined the cut-off for adverse effects as the 95th percentile, which was calculated by the model at no cadmium exposure (U-Cd = 0). Therefore, any influence of the exposure level on the reference group should have been minimized, and the potential covariates, such as age and smoking status, should have been appropriately adjusted by multiple linear regression analysis.

In the present study, estimation of BMD/BMDL was performed separately in each gender, because our previous studies in a Japanese population showed gender differences in BMD/BMDL of urinary Cd for renal tubular function [7-11].

IBM SPSS 19 J (IBM Business Analytics, Tokyo, Japan) and Microsoft Excel 2010 (Microsoft Corporation, Redmond, WA, USA) were used for the statistical analyses.

## Results

The characteristics of the participants including prevalence of those diseases increasing the risk of renal dysfunction and data on exposure and markers of renal effects grouped according to area, gender and age are shown in Table 1. The number of subjects aged 40–49 in the polluted area was greater than that of the non-polluted area. In the non-polluted area, the geometric means in all aged subjects were 0.5 μg/g Creat in men and 1.1 μg/g Creat in women for urinary Cd and 0.9 μg/L in men and 0.8 μg/L in women for blood Cd, without a significant difference between age groups. In the polluted area, the geometric means of urinary and blood Cd for all age groups were significantly greater in both genders compared with those in the non-polluted area: 6.5 μg/g Creat in men and 7.1 μg/g Creat in women for urinary Cd and 6.9 μg/L in men and 5.2 μg/L in women for blood Cd for total subjects. Urinary β2-MG and NAG were also generally greater in the polluted areas than in the non-polluted areas for both genders and all age groups. Smoking rates were greater in the polluted area compared with the non-polluted area for both genders (Table 1). Although there was no difference of prevalence of hypertension between these 2 areas, prevalence of diabetes were generally higher in the non-polluted area compared with those in the polluted area. In contrast, prevalence of nephrolithiasis was greater in the polluted area in both genders (Table 1).

**Table 1:** Characteristics of the participants and data on exposure, renal markers and prevalence of illness grouped according to area and gender

		**Non-polluted area**					**Polluted area**					**Total**				
**Age groups (yrs)**		**40-49**	**50-59**	**60-69**	**≥70**	**Total**	**40-49**	**50-59**	**60-69**	**≥70**	**Total**	**40-49**	**50-59**	**60-69**	**≥70**	**Total**
**Men**	N	9	9	12	10	40	71	52	54	53	230	80	61	66	64	270
Age (yrs)	M (SD)	44.5 (2.8)	54.6 (3.1)	64.6 (2.9)	76.4 (4.9)	61.1 (12.4)	44.1 (2.7)	52.0 (3.2)	63.7 (2.7)	75.6 (4.4)	55.6 (14.0)	44.5 (2.7)	54.2 (3.2)	63.9 (2.8)	75.7 (4.5)	56.3 (13.9)
Urinary Cd (μg/g Creat)	GM (GSD)	0.4 (2.5)	0.5 (2.1)	0.7 (1.4)	0.5 (1.8)	0.5 (1.9)	5.9 (1.8)	7.0 (2.0)	6.9 (1.7)	6.3 (2.0)	6.3 (1.9)	4.4 (2.9)	4.8 (3.3)	4.5 (2.8)	4.1 (3.2)	4.5 (2.9)
Blood cadmium (μg/L)	GM (GSD)	0.9 (2.7)	0.7 (2.5)	1.0 (1.8)	0.9 (2.0)	0.9 (2.2)	6.1 (1.9)	6.7 (1.9)	7.8 (1.8)	7.3 (1.8)	6.9 (1.9)	4.9 (2.5)	4.9 (2.8)	5.4 (2.7)	5.1 (2.7)	5.1 (2.7)
ß2-MG (μg/g Creat)	GM (GSD)	133 (2.2)	124 (3.4)	214 (3.6)	832 (3.5)	249.6 (4.0)	101 (3.6)	500 (8.9)	567 (8.6)	2221 (11.3)	443.3 (9.9)	104 (3.5)	407 (8.4)	475 (7.8)	1876 (9.8)	407.2 (9.0)
NAG (IU/g Creat)	GM (GSD)	3.3 (1.6)	5.5 (2.2)	4.7 (1.7)	8.0 (2.5)	5.2 (2.1)	4.2 (1.7)	5.3 (1.9)	6.1 (1.7)	7.9 (1.7)	5.3 (1.9)	4.1 (1.7)	5.4 (2.0)	5.8 (1.7)	7.9 (1.8)	5.3 (1.9)
Smoking habit																
Ex-smokers	%	44.4	55.6	45.5	36.4	45.0	25.4	28.8	35.2	54.7	31.2	27.5	32.8	36.9	51.6	33.0
Smokers	%	44.4	44.4	36.4	36.4	40.0	67.6	67.3	61.1	41.5	53.1	65	63.9	56.9	40.6	51.3
Prevalence of illness																
Hypertension	%	22.2	22.2	41.7	36.4	32.5	8.5	19.2	27.8	50.9	22.3	10	19.7	30.3	48.4	23.7
Diabetes	%	0.0	11.1	8.3	0.0	5.0	1.4	1.9	1.9	5.7	2.3	1.3	3.3	3.0	4.7	2.7
Nephrolithiasis	%	0.0	0.0	8.3	0.0	2.5	4.2	11.5	5.6	3.8	5.4	3.8	9.8	6.1	3.1	5.0
**Women**	N	10	16	9	6	41	108	119	96	47	370	118	135	105	53	411
Age (yrs)	M (SD)	46.3 (2.7)	54.9 (2.6)	64.2 (2.8)	77.2 (5.4)	58.1 (10.5)	44.6 (3.0)	54.2 (3.0)	64.1 (3.1)	74.8 (4.6)	53.1 (12.7)	44.7(3.0)	54.3 (2.9)	64.1 (3.1)	75.1 (4.7)	53.5 (12.6)
Urinary Cd (μg/g Creat)	GM (GSD)	0.8 (2.3)	1.3 (2.0)	1.0 (3.2)	1.3 (1.8)	1.1 (2.3)	6.5 (2.1)	7.6 (1.9)	7.5 (2.0)	6.5 (1.8)	7.0 (1.9)	5.4 (2.6)	6.1 (2.3)	6.3 (2.5)	5.4 (2.2)	5.9 (2.3)
Blood cadmium (μg/L)	GM (GSD)	0.5 (1.7)	1.0 (2.1)	0.9 (2.1)	1.1 (1.9)	0.8 (2.1)	4.5 (2.2)	5.7 (1.9)	5.7 (2.0)	5.1 (1.8)	5.2 (2.0)	3.7 (2.7)	4.6 (2.4)	4.9 (2.4)	4.3 (2.2)	4.4 (2.5)
ß2-MG (μg/g Creat)	GM (GSD)	43.9 (2.2)	160 (4.2)	260 (6.4)	1952 (8.9)	187.2 (6.6)	151.9 (6.5)	154 (4.7)	311 (8.1)	397 (7.6)	207.7 (6.6)	137 (6.3)	155 (4.6)	307 (7.9)	475 (8.0)	205.6 (6.6)
NAG (IU/g Creat)	GM (GSD)	2.7 (1.7)	5.3 (2.3)	4.6 (2.1)	10.7 (2.0)	4.8 (2.3)	4.9 (1.7)	5.6 (1.7)	6.6 (1.8)	8.6 (1.8)	5.7 (1.9)	4.6 (1.8)	5.6 (1.8)	6.4 (1.9)	8.8 (1.8)	5.6 (1.9)
Smoking habit																
Ex-smokers	%	0.0	6.3	44.4	83.3	24.4	11.1	27.7	53.1	42.6	26.4	10.2	25.2	52.4	47.2	26.2
Smokers	%	0.0	25.0	11.1	0.0	0.1	21.3	29.6	28.1	42.6	23.2	19.5	26.7	26.7	37.7	22.2
Prevalence of illness																
Hypertension	%	30	25	22.2	50	29.3	15.7	30.3	40.6	55.3	26.8	16.9	29.6	39	54.7	29.5
Diabetes	%	0.0	25.0	22.2	16.7	17.1	5.6	5.9	11.5	4.3	5.9	5.1	8.1	12.4	5.7	6.9
Nephrolithiasis	%	0.0	0.0	0.0	0.0	0.0	0.0	10.9	4.2	12.8	5.2	0.0	9.6	3.8	11.3	4.8

SD: standard deviation. GM: geometric mean. GSD: geometric standard deviation.

The levels of urinary and blood Cd were significantly greater in the polluted area than those in the non-polluted area for all age groups (P < 0.001 student’s *t*-test).

Table 2 shows the results of the multiple linear regression analysis between urinary or blood Cd and renal markers, grouped according to gender. Both urinary and blood Cd were related significantly to all of the natural log-transformed renal markers after adjusting for age and smoking habit, indicating the relevance of the dose-effect relationship between Cd exposure and renal markers. In this study, the significant regression coefficients for β2-MG were 0.06 in men and 0.05 in women for a 1 μg/g Creat increase of urinary Cd and 0.07 in both genders for a 1 μg/L increase of blood Cd. For urinary NAG, regression coefficients were 0.03 in men and 0.02 in women for 1 μg/g Creat increase of urinary Cd and 1 μg/L increase of blood Cd.

**Table 2:** Results of the multiple linear regression analysis between urinary Cd and renal markers grouped according to gender

		**Men**		**Women**	
**Renal effect markers**^**a**^	**Explanatory variables**	**B**^**b **^**(95% CI**^**c**^**)**	**P**	**B**^**b **^**(95% CI**^**c**^**)**	**P**
β2-MG (μg/g Creat)	Urinary Cd (μg/g Creat)	0.06 (0.02-0.10)	0.002	0.05 (0.02-0.08)	0.001
	Age (yrs)	0.08 (0.06-0.10)	<0.001	0.04 (0.02-0.05)	<0.001
	Smoking habit (/non-smokers)				
	Ex-smokers	0.99 (-0.01-1.99)	0.053	0.64 (0.19-1.09)	0.005
	Smokers	1.28 (0.31-2.26)	0.010	0.56 (0.11-1.00)	0.015
β2-MG (μg/g Creat)	Blood Cd (μg/L)	0.07 (0.02-0.11)	0.003	0.07 (0.03-0.11)	<0.001
	Age (yrs)	0.08 (0.06-0.10)	<0.001	0.04 (0.02-0.05)	<0.001
	Smoking habit (/non-smokers)				
	Ex-smokers	0.93 (-0.08-1.94)	0.070	0.55 (0.10-1.00)	0.017
	Smokers	1.15 (0.16-2.14)	0.023	0.41 (-0.04-0.86)	0.077
NAG (IU/g Creat)	Urinary Cd (μg/g Creat)	0.03 (0.02-0.05)	<0.001	0.02 (0.02-0.03)	<0.001
	Age (yrs)	0.02 (0.01-0.02)	<0.001	0.02 (0.01-0.02)	<0.001
	Smoking habit (/non-smokers)				
	Ex-smokers	0.18 (-0.11-0.47)	0.228	0.13 (-0.01-0.27)	0.078
	Smokers	0.12 (-0.17-0.40)	0.418	0.15 (0.00-0.29)	0.045
NAG (IU/g Creat)	Blood Cd (μg/L)	0.03 (0.01-0.04)	<0.001	0.02 (0.01-0.04)	<0.001
	Age (yrs)	0.02 (0.01-0.02)	<0.001	0.02 (0.01-0.02)	<0.001
	Smoking habit (/non-smokers)				
	Ex-smokers	0.17 (-0.13-0.47)	0.272	0.10 (-0.05-0.24)	0.202
	Smokers	0.08 (-0.22-0.37)	0.608	0.09 (-0.05-0.24)	0.210

^a^All renal markers were natural log-transformed. ^b^Regression coefficients. ^c^95% confidence interval.

Table 3 shows the BMD and BMDL of urinary and blood Cd for renal markers in all subjects and in the subjects without nephrolithiasis. The BMDL/BMD values of urinary Cd (μg/g Creat) for renal effect markers in all subjects were 6.9/11.3 for β2-MG and 4.4/5.8 for NAG in men and 8.1/12.9 for β2-MG and 6.1/8.4 for NAG in women. These calculated BMDL values for urinary NAG, but not for β2-MG, were less than the geometric mean urinary Cd in the polluted area, 6.5 in men and 7.1 μg/g Creat in women (Table 1). The BMDL/BMD of blood Cd (μg/L) for renal markers were 6.2/10.2 for β2-MG and 5.0/7.4 for NAG in men and 5.9/9.1 for β2-MG and 5.8/8.7 for NAG in women. These calculated BMDLs for both two renal markers in men, but not in women, were less compared with the geometric mean blood Cd in the polluted area, 6.9 in men and 5.2 μg/L in women (Table 1).

**Table 3:** Benchmark doses of urinary and blood cadmium for renal markers calculated using the hybrid approach in all subjects and subjects without nephrolithiasis

	**Men**		**Women**	
**Renal effect markers**^**a**^	**Estimated cut-off value**^**b**^	**BMDL (BMD)**	**Estimated cut-off value**^**b**^	**BMDL (BMD)**
Urinary Cd (μg/g Creat)				
*All subjects*				
β2-MG (μg/g Creat)	2004	6.9 (11.3)	1815	8.1 (12.9)
NAG (IU/g Creat)	9.4	4.4 ( 5.8)	11.2	6.1 ( 8.4)
*Subjects without nephrolithiasis*				
β2-MG (μg/g Creat)	1853	6.9 (11.7)	1767	8.2 (13.2)
NAG (IU/g Creat)	9.2	4.2 ( 5.6)	11.1	6.0 ( 8.3)
Blood Cd (μg/L)				
*All subjects*				
β2-MG (μg/g Creat)	1694	6.2 (10.2)	1664	5.9 ( 9.1)
NAG (IU/g Creat)	9.8	5.0 ( 7.4)	11.6	5.8 ( 8.7)
*Subjects without nephrolithiasis*				
β2-MG (μg/g Creat)	1947	6.4 (11.3)	1803	5.8 (9.0)
NAG (IU/g Creat)	10.4	5.4 (8.4)	12.1	5.5 (8.3)

^a^All renal markers were natural log-transformed. ^b^Cut-off values are adjusted to mean age and non-smoker.

There were 15 cases in men and 23 cases in women of nephrolithiasis.

In addition, the BMD and BMDL values of urinary Cd in women were greater than those in men, but those of blood Cd in women were less than those in men, suggesting gender differences. Moreover, to eliminate the influence of nephrolithiasis on renal function, the BMD and BMDL of urinary and blood Cd for renal markers were recalculated in the subjects without nephrolithiasis, but these values were similar to the BMD and BMDL analyzed in all subjects (Table 3).

## Discussion

Recently, the reference level of urinary Cd for renal tubular effects has been reduced to prevent the adverse health effects of low level Cd exposure in the general population. The Joint FAO/WHO Expert Committee on Food Additives (JECFA) determined a provisional tolerable monthly intake (PTMI) of 25 μg/kg body weight [28], which corresponds to the provisional tolerable weekly intake (TWI) of 5.8 μg/kg body weight, reduced from the previous provisional TWI of 7 μg Cd/kg body weight [29]. Furthermore, the European Food Safety Authority (EFSA) performed a meta-analysis applying the BMD approach to the dose–response relationship between urinary Cd and β2-MG in various previous studies [30]. The calculated reference point for urinary Cd was 1 μg/g Creat and it was converted into dietary exposure based on the one-compartment model [31].

In the present study, the calculated BMDLs of urinary Cd for the renal markers were in the range of 4.4 - 6.9 μg/Creat in men and 6.1 - 8.1 μg/g Creat in women, which are equal to 33–54 percentile of urinary Cd in men and 48–55 percentile in women in the polluted area. These results indicate that urinary Cd levels of 46 - 77% men and 45-52% women in the polluted area are more than the BMDLs. Similarly, the calculated BMDLs of blood Cd for the renal markers were in the range of 5.0 - 6.2 μg/L in men and 5.8 - 5.9 μg/L in women, suggesting that 42 - 61% men and 45 - 47% women showed greater blood Cd levels than BMDLs in the polluted area. Because of the high prevalence of nephrolithiasis related to increased urinary Cd [24] which may influence renal function in the polluted area, BMDLs of urinary or blood Cd for the renal markers were recalculated in the subjects without nephrolithiasis. However, BMDLs of urinary or blood Cd for renal markers were quite similar to those values in all subjects, suggesting the high prevalence of nephrolithiasis had no effect on the BMDLs in these populations. We also calculated the BMDLs of urinary Cd for renal markers with other confounding factors, such as pH and calcium concentration in urine, but very little difference was observed (data not shown). Therefore, taken together, more than 40% of the residents aged more than 40 years old in Mae Sot, Thailand were suspected to be at risk of adverse renal effects induced by Cd. Efficient measures to decrease Cd exposure are necessary for the inhabitants living in these polluted areas in Thailand.

Previously, the BMD method has been applied to estimate the reference point for Cd-related renal dysfunction using urinary Cd, not blood Cd [7-10,32,33], rice Cd concentration [34], and lifetime Cd intake [35,36]. However, in most of these studies, estimation of BMD and BMDL was performed using Benchmark Dose Software (BMDS) developed by the United States Environmental Protection Agency (U.S. EPA) [5]. The subjects were categorized according to their exposure level, and the response was dichotomized based on the renal effect marker due to the specification of BMDS. However, according to the hybrid approach, benchmark dose is not dependent upon categorization of exposures, the number of categories, or the dose-intervals, which have marked effects on the results by decreasing statistical power [37]. We then applied the hybrid approach in several recent Japanese studies and observed significant dose–response relationships between urinary Cd and renal tubular effect markers in the non-polluted subjects with 1.1 μg/g Creat of mean urinary Cd in men and 2.2 μg/g Creat in women [15,16] and in the polluted subjects with 3.2 μg/g Creat of mean urinary Cd in men and 4.3 μg/g Creat in women [17] in Japan. Smoking rates for men and women in Japan were similar for men and less for women than those of the MaeSot population, Thailand. In these Japanese studies, the estimated BMDLs of urinary Cd for renal tubular markers ranged from 0.6 - 4.1 μg/g Creat and 0.6 - 3.7 μg/g Creat in men and women, respectively after adjusting for age and smoking status [15-17]. In another study conducted in 17,375 adult women living in 16 Cd non-polluted areas in Japan, significant relationships were observed between urinary Cd and β2-MG in 15 areas, with the estimated BMDLs of urinary Cd for β2-MG ranging from 0.9 - 3.8 μg/g Creat with a median of 1.4 μg/g Creat [18]. However, in the present study, the calculated BMDLs were greater than those in the Japanese studies. One reason may be that the present subjects in the polluted area were selected residents because of high urinary Cd (≥5 μg/g Creat) in the 2004–5 survey. Another reason might be a high prevalence of another disease than Cd nephropathy that increases urinary β2-MG and NAG in the present area. However, as described in an earlier part of the discussion section, nephrolithiasis which is common in the Thai population, was suspected to be a confounding factor affecting the relationship between urinary Cd and renal effects, but recalculated BMDLs were not much different after elimination of the nephrolithiasis cases. Moreover, diabetes is well-known to increase urinary β2-MG, but the prevalence of diabetes was less than that in the polluted area than those in the non-polluted area (2.3 for men and 5.9 for women in the Cd-polluted area compared with 5.0 for men and 16.7 for women in the non-polluted area), suggesting no influence of diabetes in the polluted area for increasing renal dysfunction indicated by urinary β2-MG. In addition, Caumont et al. [38] reported that the BMDL of urinary Cd was less in ever smokers compared with never smokers in Belgian Cd-exposed workers. Therefore, particularly in Thai residents with a high rate of smoking, elimination of the influence of smoking to estimate BMDL was important, and the hybrid approach was applied to adjust for smoking status and age in the present study.

Although the BMDL of urinary Cd has been established for renal effects, BMDL estimation based on the hybrid approach has not been conducted for blood Cd in previous studies. One potential reason is the nature of blood Cd. It is well known that urinary Cd is mainly influenced by the body burden of Cd and is proportional to the concentration in the kidney [6,20]. In contrast, blood Cd has been considered the most valid marker of recent exposure [6,20]. The half-life of blood Cd displays a fast component of 3 to 4 months and a slow component of approximately 10 years [39]. However, the high correlation between urinary and blood Cd [6,20,22] indicates that urinary Cd is thought to be a good estimate of cadmium body burden in environmentally exposed populations whose Cd exposure is consistent. Because such an influential acute Cd exposure was less likely in the present area because of the prohibition of rice farming at the time the study was performed, we estimated BMDLs of blood Cd as an index of Cd exposure in the present study.

Furthermore, blood Cd was of little relevance to the Cd concentration in the kidneys. Therefore, a significant relationship between blood Cd and renal tubular markers found in the present study demonstrated the relationship between Cd exposure and renal tubular dysfunction. We consider this point is an important notable feature of the present study. Moreover, we defined the cut-off for adverse effects as the 95th percentile, calculated by the model at no cadmium exposure (urinary Cd = 0) in the study population. Therefore, this approach did not require the actual reference population to determine the cut-offs, which may have a large influence on the estimation of BMDL by the classical method using prevalence [19]. Therefore in the present study, any influence of the exposure level on the reference group was minimized. Additionally, the potential covariates, such as age and smoking status, were adjusted appropriately by multiple linear regression analysis in the present study. Although we believe that further estimation and discussion of the BMDL of blood Cd for renal dysfunction is necessary, we conclude that the reliability of BMDL in the present study was increased considerably, because BMDL that was estimated using blood Cd as an exposure marker was consistent with that of urinary Cd.

## Conclusion

Estimations of BMDL for renal effects of both urinary Cd and blood Cd as exposure markers were useful to increase analytical reliability in a Thai population. The BMDLs of urinary and blood Cd for renal effects were estimated to be 4.4 - 8.1 μg/g Creat and 5.0 - 6.2 μg/L, respectively, in the Thai population aged ≥ 40. These BMDLs suggest that more than 40% of the residents were at a high risk of renal effects induced by Cd exposure, because these values were in the 33–55 percentiles of urinary and 39–58 percentiles of blood Cd levels in the exposed subjects.

## Competing interests

All authors have approved the final version of the manuscript for publication, and declared all relevant competing interests. There is no competing interest for this paper.

## Authors’ contributions

HN, and MN participated in the design and coordination of the study. WR, KNo, and WS performed the preparation of survival data and data set creation. SM, YM, KNa, and WS conducted the survey for baseline data collection. YS performed the statistical analysis. MN and YS prepared the draft for the manuscript. All authors read and approved the final manuscript.

## Pre-publication history

The pre-publication history for this paper can be accessed here:

http://www.biomedcentral.com/1471-2458/14/702/prepub
